# Metabolically healthy and unhealthy obesity phenotypes in the general population: the FIN-D2D Survey

**DOI:** 10.1186/1471-2458-11-754

**Published:** 2011-10-01

**Authors:** Pia Pajunen, Anna Kotronen, Eeva Korpi-Hyövälti, Sirkka Keinänen-Kiukaanniemi, Heikki Oksa, Leo Niskanen, Timo Saaristo, Juha T Saltevo, Jouko Sundvall, Mauno Vanhala, Matti Uusitupa, Markku Peltonen

**Affiliations:** 1 Diabetes Prevention Unit, Division of Welfare and Health Promotion, National Institute for Health and Welfare, Helsinki, Finland; 2 Department of Medicine, Division of Diabetes, University of Helsinki, Helsinki, Finland; 3 Minerva Medical Research Institute, Helsinki, Finland; 4 Department of Internal Medicine, South Ostrobothnia Central Hospital, Seinäjoki, Finland; 5 Institute of Health Sciences (General Practice), University of Oulu, Finland; 6 Unit of General Practice, Oulu University Hospital and Health Centre of Oulu, Oulu, Finland; 7 Tampere University Hospital, Tampere, Finland; 8 Department of Medicine/Diabetology and Endocrinology, Kuopio University Hospital, Kuopio, Finland; 9 Finnish Diabetes Association, Tampere, Finland; 10 Department of Medicine, Central Finland Central Hospital, Jyväskylä, Finland; 11 Disease Risk Unit, Department of Chronic Disease Prevention, National Institute for Health and Welfare, Helsinki, Finland; 12 School of Medicine, Unit of Primary Health Care, University of Eastern Finland, Kuopio, Finland; 13 Unit of Family Practice, Central Hospital of Central Finland, Jyväskylä, Finland; 14 Institute of Public Health and Clinical Nutrition, Clinical Nutrition, University of Eastern Finland, and Research Unit, Kuopio University Hospital, Kuopio, Finland

## Abstract

**Background:**

The aim of this work was to examine the prevalence of different metabolical phenotypes of obesity, and to analyze, by using different risk scores, how the metabolic syndrome (MetS) definition discriminates between unhealthy and healthy metabolic phenotypes in different obesity classes.

**Methods:**

The Finnish type 2 diabetes (FIN-D2D) survey, a part of the larger implementation study, was carried out in 2007. The present cross-sectional analysis comprises 2,849 individuals aged 45-74 years. The MetS was defined with the new Harmonization definition. Cardiovascular risk was estimated with the Framingham and SCORE risk scores. Diabetes risk was assessed with the FINDRISK score. Non-alcoholic fatty liver disease (NAFLD) was estimated with the NAFLD score. Participants with and without MetS were classified in different weight categories and analysis of regression models were used to test the linear trend between body mass index (BMI) and various characteristics in individuals with and without MetS; and interaction between BMI and MetS.

**Results:**

A metabolically healthy but obese phenotype was observed in 9.2% of obese men and in 16.4% of obese women. The MetS-BMI interaction was significant for fasting glucose, 2-hour plasma glucose, fasting plasma insulin and insulin resistance (HOMA-IR)(p < 0.001 for all). The prevalence of total diabetes (detected prior to or during survey) was 37.0% in obese individuals with MetS and 4.3% in obese individuals without MetS (p < 0.001). MetS-BMI interaction was significant (p < 0.001) also for the Framingham 10 year CVD risk score, NAFLD score and estimated liver fat %, indicating greater effect of increasing BMI in participants with MetS compared to participants without MetS. The metabolically healthy but obese individuals had lower 2-hour postload glucose levels (p = 0.0030), lower NAFLD scores (p < 0.001) and lower CVD risk scores (Framingham, p < 0.001; SCORE, p = 0.002) than normal weight individuals with MetS.

**Conclusions:**

Undetected Type 2 diabetes was more prevalent among those with MetS irrespective of the BMI class and increasing BMI had a significantly greater effect on estimates of liver fat and future CVD risk among those with MetS compared with participants without MetS. A healthy obese phenotype was associated with a better metabolic profile than observed in normal weight individuals with MetS.

## Background

Obesity is a major contributor to the global epidemic of type 2 diabetes [1] to fatty liver disease [2] and to cardiovascular diseases (CVD) [3]. Worldwide, at least 300 million individuals are clinically obese [4] and in Finland, out of those aged 25-74 years, 25% are obese and over half are overweight [5].

Metabolic abnormalities which are usually associated with obesity, do not, however, affect all obese people. Approximately 10-25% of obese people [6] and a fraction of morbidly obese individuals [7] are not affected by metabolic disturbances [8-11]. These "metabolically healthy but obese" subjects are insulin sensitive, have normal blood pressure, a favorable lipid profile, a lower proportion of visceral fat, less liver fat and a normal glucose metabolism despite having an excessive amount of body fat [9-17].

On the other hand, a subset of normal weight individuals suffer from metabolic disturbances that are characteristic of obesity [18]. These individuals are called "metabolically obese, normal weight individuals" [19,20]. Thus, obesity consists of different subtypes with different metabolic profiles. Although these phenotypes have been recognized by the scientific community, not much data exists on the subject. It has been suggested that metabolically healthy obesity may have a less adverse metabolic profile and outcome than normal weight individuals with metabolic syndrome (MetS). However, there are only a few studies comparing these phenotypes and giving the true estimation of characteristics of these phenotypes in the general population [8,19].

In this study, we examine the prevalence of different metabolic phenotypes of obesity, especially the "metabolically healthy but obese" phenotype, and analyze, by using different risk scores, how the MetS definition discriminates between unhealthy and healthy metabolic phenotypes in different obesity classes in a large population-based cohort of 2,849 individuals.

## Methods

### FIN-D2D survey

As part of evaluation of the implementation project of the national type 2 diabetes prevention programme (FIN-D2D), a survey was carried out in three hospital districts in Finland between October and December 2007 [21]. A random sample of 4,500 subjects aged 45-74 years, stratified according to gender, 10-year age groups (45-54, 55-64, and 65-74 years), and the three geographical areas, was selected from the National Population Register. The overall participation rate was 64%. In addition, 26 subjects were excluded from the present analyses due to missing data on variables needed for defining the MetS (n = 17) or BMI (n = 19). The total number of individuals included was thus 2,849 (63% of the original sample). The study protocol was approved by the Ethical Committee of the Hospital District of Helsinki and Uusimaa and all participants gave their written and informed consent.

### Clinical examination

Subjects were invited by mail to a clinical examination. Together with the invitation, they also received a self-administered questionnaire on medical history and health behaviour. They were asked to complete the questionnaire at home, and bring it with them to the health examination, which was carried out according to the WHO MONICA project protocol [22]. At the study site, trained nurses measured height, weight and waist circumference, as well as BP using a standardized protocol. Height was measured to the nearest 0.1 cm. Body weight of the participants wearing usual light indoor clothing without shoes was measured with a 0.1 kg precision. Blood pressure was measured twice in a sitting position after a minimum of five minutes of acclimatization and before blood sampling using a mercury sphygmomanometer. The mean of the two blood pressure measurements was used in the analyses.

### Classification of obesity and the MetS

BMI was calculated as weight (kg) divided by height × height (m^2^). Overweight and obesity were defined as BMI 25-29 kg/m^2 ^and ≥ 30 kg/m^2^, respectively. The MetS was defined according to the Harmonization definition [23], which requires three or more of the following five components: large waist circumference (≥ 94 cm in men and ≥ 80 cm in women), hypertriglyceridemia (≥ 1.7 mmol/l), HDL cholesterol level < 1.0 mmol/l in men or < 1.3 mmol/l in women, elevated blood pressure (systolic ≥ 130 mmHg and/or diastolic ≥ 85 mmHg and/) or antihypertensive drug treatment or history of hypertension, elevated fasting plasma glucose ≥ 5.6 mmol/l or drug treatment.

### Glucose tolerance status

The glucose tolerance status was classified according to the WHO 1999 criteria [24]. Individuals who already had diagnosed type 2 diabetes were not included in the OGTT and were classified as known diabetic participants. Individuals who had not been diagnosed as diabetic, but who had a fasting plasma glucose level of ≥ 7.0 mmol/l or 2 h plasma glucose ≥ 11.1 were classified as having screen-detected type 2 diabetes. The known diabetic individuals and the screen-detected diabetic individuals were combined to create a group defined as total type 2 diabetics.

### Biochemical measurements

All assays were performed at the Disease Risk Unit of the National Institute for Health and Welfare, Helsinki, using Architect ci8200 analyzer (Abbott Laboratories, Abbott Park, IL, US). Plasma glucose was determined with a hexokinase method (Abbott Laboratories, Abbott Park, IL) and serum insulin with a chemiluminescent microparticle immunoassay (Abbott Laboratories, Abbott Park, IL, US). Serum total and HDL cholesterol, and triglyceride concentrations were measured with enzymatic kits from Abbott Laboratories (Abbott Park, IL, US). The lipoproteins apoA1 and apoB were determined with an immunoturbidimetric method (Abbott Park, IL, US). The concentrations of LDL cholesterol were calculated using the Friedewald formula [25]. Serum ALT, AST, and γGT concentrations were determined using photometric IFCC (International Federation of Clinical Chemistry) methods (Abbott Laboratories, Abbott Park, IL, US). High-sensitivity C-reactive protein (hsCRP) was measured with an immunoturbidimetric method (Sentinel Diagnostics, Milano, Italy).

### Risk scores

Cardiovascular risk was estimated with the Framingham [26] and SCORE risk scores [27]. Diabetes risk was assessed with the FINDRISK score [28]. Non-alcoholic fatty liver disease (NAFLD) was estimated with the NAFLD score [29].

### Lifestyle definitions

The average daily alcohol consumption (g/d) was calculated from the self-reported number of drinks taken during the past week.

The estimation of fruit and vegetable consumption was derived from the question: "How often do you eat fruit, vegetables and brown bread (rye- or whole-grain bread)?" The possible answers were: 1) every day, and 2) not every day. Fruit and vegetable consumption was considered scarce if it did not occur daily.

Leisure time physical activity was estimated with the question: "How much do you exercise or exert yourself physically in your leisure time?" Endurance training such as jogging or swimming at least 3 hours per week was classified as "active". Endurance training less than 3 hours per week was considered "inactive".

Weight change during the past year was ascertained from the question:" How much does your weight differ from the weight you had one year ago?" The average amount of sleep was calculated from the question:" How many hours do you sleep on average each night?"

### Statistical methods

Mean values with standard deviations and proportions were used to describe the characteristics of different obesity subgroups. For continuous variables, analysis of covariance (ANCOVA) was used to test the linear trend between BMI and various characteristics in individuals with and without MetS, respectively. Similarly, logistic regression models were used for analyses of dichotomous variables. ANCOVA and logistic regression models were further used to test the interaction between BMI and MetS when considering the associations. All p-values are two-sided and p < 0.05 was considered as statistically significant. Statistical analyses were carried out using the Stata statistical package 10.1 (Stata-Corp. 2007. Stata Statistical Software: Release 10.1. College Station, TX; StataCorp LP).

## Results

**Figure 1:**
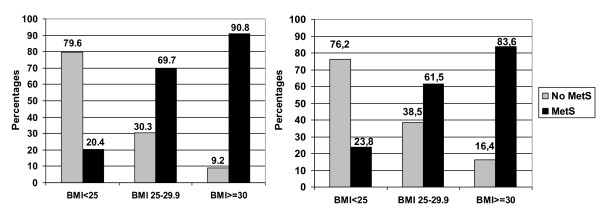
**Prevalence of persons with and without MetS within each BMI category among men (left panel) and among women (right panel) (total 100% within the BMI class)**.

A metabolically healthy but obese phenotype was observed in 9.2% of obese men and in 16.4% of obese women (Figure 1). Among all participants, the prevalence of healthy obesity was 2.0% among men and 4.5% among women. Of the normal weight individuals, 20.4% of men and 23.8% of women had the MetS (Figure 1). MetS increased with age in both sexes (data not shown).

Table 1 presents the distribution of the MetS definition components by obesity class in persons with and without MetS. Table 2 shows CVD risk factors, parameters related to glucose metabolism and liver function as well as lifestyle factors by obesity class in persons with and without MetS. Overall, in all weight categories (normal weight BMI < 25, overweight BMI 25-29.9, obese BMI ≥ 30) individuals with MetS had a more adverse metabolic profile and greater cardiovascular and diabetes risk scores compared with the individuals without MetS (Tables 1 and 2).

**Table 1:** Basic characteristics and components of the metabolic syndrome in individuals with and without metabolic syndrome in different BMI classes

	No MetS				MetS				
	**BMI < 25** **No MetS** **(n = 712)**	**BMI 25-29.9** **No MetS** **(n = 418)**	**BMI ≥ 30** **No MetS** **(n = 94)**	**P** **trend**	**BMI < 25** **MetS** **(n = 205)**	**BMI 25-29.9** **MetS** **(n = 811)**	**BMI ≥ 30** **MetS** **(n = 609)**	**P** **trend**	**P** **MetS/** **BMI** **Interaction**

Population prevalence, %	25.0	14.7	3.3		7.2	28.5	21.3		

Proportion of men, %	43.8	48.6	28.7		39.0	57.6	44.2		

Age (yr)	57.8 ± 8.5	57.6 ± 8.3	58.5 ± 8.2	0.801	60.3 ± 8.1	61.4 ± 7.8	61.3 ± 8.3	0.356	0.593

Height (cm)	168.7 ± 8.7	168.7 ± 8.6	165.8 ± 9.6	0.053	169.1 ± 9.3	170.3 ± 9.2	167.7 ± 9.0	< 0.001	< 0.001

Weight (kg)	64.4 ± 8.7	76.6 ± 8.4	91.4 ± 12.6	< 0.001	68.1 ± 8.5	80.1 ± 9.6	96.2 ± 15.0	< 0.001	0.003

**MetS components**									

Waist (cm)	81.2 ± 7.5	91.8 ± 7.0	105.1 ± 9.7	< 0.001	88.4 ± 6.7	97.4 ± 7.5	111.1 ± 11.2	< 0.001	0.205

BMI (kg/m^2^)	22.5 ± 1.7	26.9 ± 1.3	33.2 ± 3.1	< 0.001	23.7 ± 1.1	27.5 ± 1.3	34.1 ± 4.2	< 0.001	< 0.001

SBP (mmHg)	128.8 ± 18.1	127.2 ± 15.5	130.1 ± 16.7	0.589	139.2 ± 16.7	143.3 ± 17.5	144.7 ± 17.3	< 0.001	0.006

DBP (mmHg)	78.1 ± 9.3	78.8 ± 8.4	79.2 ± 7.7	0.077	82.8 ± 8.8	83.7 ± 9.2	84.8 ± 9.5	0.003	0.424

FPG (mmol/l)	5.8 ± 0.88	5.8 ± 0.94	5.8 ± 1.4	0.585	6.2 ± 0.85	6.5 ± 1.2	6.7 ± 1.4	< 0.001	< 0.001

Triglycerides (mmol/l)	0.96 ± 0.34	1.1 ± 0.34	1.2 ± 0.35	< 0.001	1.4 ± 0.68	1.6 ± 0.97	1.8 ± 1.0	< 0.001	< 0.001

HDL cholesterol (mmol/l)	1.6 ± 0.34	1.5 ± 0.31	1.5 ± 0.21	< 0.001	1.4 ± 0.38	1.4 ± 0.33	1.3 ± 0.27	< 0.001	0.029

BMI, body mass index; FPG, fasting plasma glucose; HDL cholesterol, high-density lipoprotein cholesterol; DBP, diastolic blood pressure;

SBP, systolic blood pressure. P values adjusted for age and sex (except p for age which is adjusted for sex only).

**Table 2:** Characteristics and laboratory results in individuals with and without metabolic syndrome in different BMI classes

	No MetS				MetS				
	**BMI < 25** **(n = 712)**	**BMI 25-29.9** **(n = 418)**	**BMI ≥ 30** **(n = 94)**	**P** **trend**	**BMI < 25** **(n = 205)**	**BMI 25-29.9** **(n = 811)**	**BMI ≥ 30** **(n = 609)**	**P** **trend**	**P for MetS/BMI** **Interaction**

**Glucose metabolism**									

2-hour plasma glucose (mmol/l)	6.0 ± 2.1	6.3 ± 1.8	6.5 ± 2.1	0.013	7.1 ± 2.5	7.6 ± 2.7	8.6 ± 3.4	< 0.001	< 0.001

fP-insulin (mU/l)	5.1 ± 4.4	6.0 ± 2.5	8.5 ± 4.2	< 0.001	6.7 ± 4.1	9.8 ± 22.0	14.7 ± 22.6	< 0.001	< 0.001

HOMA-IR	1.4 ± 2.5	1.6 ± 0.76	2.3 ± 2.4	0.001	1.8 ± 1.1	3.3 ± 15	4.7 ± 8.7	< 0.001	< 0.001

FINDRISK diabetes risk score (points)	6.6 ± 3.4	9.8 ± 3.4	13 ± 3.1	< 0.001	10.2 ± 4.0	12.5 ± 3.9	16 ± 4.1	< 0.001	0.518

Total T2DM, %	5.1	3.8	4.3	0.490	14.4	21.3	37.0	< 0.001	< 0.001

Previously diagnosed T2DM, %	1.7	0.7	0	0.064	6.4	6.9	15.5	< 0.001	0.002

T2DM, undetected prior survey, %	3.4	3.1	4.3	0.800	7.9	14.4	21.5	< 0.001	0.075

**CVD risk**									

Cholesterol (mmol/l)	5.4 ± 0.86	5.5 ± 0.90	5.6 ± 0.98	0.001	5.6 ± 1.1	5.4 ± 1.0	5.4 ± 1.1	0.019	< 0.001

LDL cholesterol (mmol/l)	3.3 ± 0.76	3.5 ± 0.80	3.6 ± 0.87	< 0.001	3.5 ± 0.89	3.3 ± 0.89	3.3 ± 0.96	0.001	< 0.001

Apolipoprotein A1 (g/l)	1.7 ± 0.26	1.6 ± 0.24	1.6 ± 0.19	< 0.001	1.6 ± 0.30	1.5 ± 0.26	1.5 ± 0.23	< 0.001	0.985

Apolipoprotein B (g/l)	0.90 ± 0.19	0.97 ± 0.19	0.98 ± 0.19	< 0.001	1.0 ± 0.23	1.0 ± 0.23	1.0 ± 0.24	< 0.001	< 0.001

hsCRP (mg/l)	1.3 ± 3.8	2.5 ± 8.0	3.1 ± 4.6	< 0.001	3.8 ± 16	2.5 ± 5.0	4.4 ± 8.5	0.118	0.713

Framingham 10 yr CVD (%)	11.9 ± 11.1	11.3 ± 8.4	10.4 ± 8.6	0.067	18.7 ± 13.9	22.8 ± 15	25.1 ± 17.2	< 0.001	< 0.001

SCORE 10 yr fatal CVD (%)	4.5 ± 6.1	3.9 ± 4.3	3.6 ± 4.8	0.002	5.8 ± 5.9	7.2 ± 6.5	6.4 ± 5.8	0.560	0.074

**Liver**									

Serum ALT (U/l)	21.1 ± 12.8	24.4 ± 10.6	24.6 ± 12.5	< 0.001	24.7 ± 15.4	28.1 ± 15.6	32.9 ± 24.9	< 0.001	0.033

Serum AST(U/l)	24.3 ± 12.4	24.4 ± 7.4	25.6 ± 9.4	0.145	26.1 ± 13.0	26.8 ± 10.7	39.6 ± 17.7	< 0.001	0.045

Serum γGT (U/l)	26.9 ± 27.8	32.0 ± 28.1	33.0 ± 31.8	0.001	40.9 ± 117.5	40.5 ± 45.7	49.5 ± 66.6	0.065	0.636

Alcohol (g/d)	8.2 ± 13	7.6 ± 12	5.8 ± 11	0.168	8.6 ± 24	8.6 ± 14	8.0 ± 13	0.860	0.522

NAFLD score	-2.0 ± 0.96	-1.9 ± 0.57	-1.5 ± 0.84	< 0.001	-0.45 ± 1.0	0.021 ± 3.5	0.97 ± 3.6	< 0.001	< 0.001

Estimated liver fat (%)	2.1 ± 1.1	2.2 ± 0.69	2.7 ± 1.0	< 0.001	4.9 ± 2.2	5.6 ± 3.4	7.8 ± 5.1	< 0.001	< 0.001

**Lifestyle**									

Not eating fruits/vegetables daily, %	18.3	20.0	18.1	0.535	20.2	23.9	19.8	0.548	0.404

Active leisure time physical activity, %	30.9	27.6	14.1	0.002	20.6	19.3	12.1	< 0.001	0.836

Weight change during past year (kg)	-0.31 ± 0.46	0.43 ± 3.8	0.56 ± 6.9	0.015	0.2 ± 0.40	-0.03	-0.16 ± 5.7	0.588	0.108

Hours slept per night (hours)	7.2 ± 0.99	7.3 ± 1.0	7.4 ± 1.1	0.453	7.4 ± 1.1	7.3 ± 1.1	7.3 ± 1.2	0.795	0.449

Currently smoking, %	18.5	12.8	8.6	0.002	16.3	16.7	10.1	0.002	0.470

ALT: alanine aminotransferase; AST: aspartate aminotransferase; Framingham 10 yr CVD, Framingham 10-year risk score for fatal coronary events; HDL cholesterol, high-density lipoprotein cholesterol; HOMA-IR, homeostasis model assessment of insulin resistance; hsCRP, high-sensitivity C-reactive protein; NAFLD: non-alcoholic fatty liver disease; SCORE 10 yr fatal CVD, SCORE risk score 10-year risk score for fatal coronary events T2DM, type 2 diabetes mellitus; γGT: gamma glutamyltransferase. P values adjusted for age and sex.

Fasting plasma glucose levels were not modified by increasing BMI among individuals without MetS (p = 0.589 for trend, Table 1), but 2-hour plasma glucose was (p = 0.013 for trend, Table 2). Among participants with MetS, there was a significant trend for both higher fasting and 2-hour plasma glucose levels with increasing BMI (p < 0.001 for both, Tables 1 and 2). MetS-BMI interaction was significant for 2-hour plasma glucose, fasting plasma insulin and HOMA-IR (< 0.001 for all) (Table 2). The OGTT revealed a significantly higher proportion of previously undetected type 2 diabetes among those with MetS than among those without MetS irrespective of the BMI class (7.9% vs. 3.4%, p = 0.006 in BMI < 25 class, 14.4% vs. 3.1%, p < 0.001 in BMI 25-29.9 class and 21.5% vs. 4.3% in BMI ≥ 30 class). The prevalence of total diabetes (detected prior to or during survey) was 37.0% in obese individuals with MetS and 4.3% in obese individuals without MetS (p < 0.001). MetS-BMI interaction for the FINDRISK score was not significant whereas the Framingham 10 year CVD risk score was significantly higher in those with the MetS irrespective of the BMI class (Table 2).

Increasing BMI had a greater effect on ALT (MetS-BMI interaction p = 0.033), AST (MetS-BMI interaction p = 0.045), NAFLD score (MetS-BMI interaction p < 0.001) and estimated liver fat % (MetS-BMI interaction p < 0.001) in those with MetS compared with those without MetS (Table 2).

Leisure time physical activity diminished with increasing BMI class irrespective of MetS classification (Table 2). Leisure time physical activity did not differ between metabolically healthy and metabolically abnormal obese individuals (14.1% vs. 12.1%, p = 0.591). There were no differences in lifestyle variables, i.e. the daily consumption of fruits and vegetables, daily length of sleep, cigarette smoking, or reported alcohol consumption between the individuals with and without MetS.

The survey included 205 individuals (80 men and 125 women) of normal weight who had MetS and 94 (27 men and 67 women) obese individuals without MetS. The metabolically healthy but obese individuals had lower systolic and diastolic blood pressure levels than normal weight individuals with MetS (139.2 ± 16.9 vs. 130.1 ± 16.7, p < 0.001 and 82.8 ± 8.8 vs. 79.2 ± 7.7, p = 0.0007). The metabolically healthy but obese individuals had lower 2-hour postload glucose levels (6.5 ± 2.1 vs. 7.1 ± 2.5 mmol/l, p = 0.0030) than normal weight individuals with MetS. There was no difference in cholesterol or LDL-cholesterol levels but the metabolically healthy but obese individuals had lower triglyceride (1.2 ± 0.35 vs. 1.4 ± 0.68 mmol/l, p = 0.005) and higher HDL cholesterol levels (1.5 ± 0.21 vs. 1.4 ± 0.38 mmol/l, p = 0.007) than the normal weight subjects with the MetS. No difference was observed in the high-sensitivity CRP or liver enzyme values (data not shown). The metabolically healthy but obese individuals had higher scores in the FINDRISK diabetes risk test (13.3 ± 3.1 vs. 10.2 ± 4.0 points, p < 0.001), but lower prevalence of current type 2 diabetes than the normal weight subjects with MetS. The estimated 10-year fatal CVD risk (Framingham 18.7% vs. 10.4%, p < 0.001 and SCORE 5.8 vs. 3.6%, p = 0.002) was higher in the normal weight individuals with MetS than in metabolically healthy but obese individuals. The NAFLD score and estimated liver fat percentage (2.7 ± 1.0 vs. 4.9 ± 2.2, p < 0.001) were lower in the metabolically healthy but obese individuals than in normal weight individuals with MetS.

## Discussion

In this study we used the most recent criteria of the MetS [23] to identify metabolically healthy obese individuals and normal weight individuals with MetS. Among the Finnish population aged 45-74 years, the prevalence of the metabolically healthy but obese phenotype was 2.0% among men and 4.5% among women. Of the obese, about one tenth had the metabolically healthy phenotype. As there are currently no international unified criteria for defining healthy obesity, it is difficult to compare these results with the studies from other countries. Indeed, the prevalence estimates of the healthy obese phenotype vary considerably from 3.3% to 43% depending on the criteria used [6,8,14,30-34].

The clinical value, biological basis and usefulness of the MetS has been severely debated [35]. In the present study, the MetS definition discriminated well between unhealthy and healthy metabolic phenotypes in different obesity classes beyond those included in the MetS criteria. Among those with the Mets, the OGTT which was performed as part of the survey revealed a significantly higher proportion of previously undetected type 2 diabetes irrespective of BMI class. The MetS-BMI interaction was significant for fasting glucose, 2-hour plasma glucose, fasting plasma insulin and HOMA-IR, indicating that the metabolic consequences of obesity seem to be more adverse among individuals with MetS. Furthermore, increasing BMI had a significantly greater effect on estimates of liver fat among those with the MetS compared with participants without MetS. The average NAFLD liver fat score was lower in those without MetS irrespective of BMI class. Not surprisingly, NAFLD has previously been shown to predict type 2 diabetes independent of obesity [2].

In accordance with earlier data from the US [8], the metabolically healthy but obese phenotype was associated with an overall better metabolic profile than observed in normal weight individuals with MetS. Obese individuals without MetS had lower fasting plasma glucose and 2-hour postload glucose levels than normal weight individuals with MetS. In addition, they had a better lipid profile and lower CVD risk scores, less estimated liver fat and less often previously undetected diabetes compared with normal weight individuals with the MetS. In clinical work, it is thus important not only to estimate the degree of obesity but also the presence of metabolical abnormalities which are present in a significant proportion of the normal weight individuals.

Some studies from other countries have suggested that a metabolically healthy but obese phenotype would be associated with decreased risk of nonfatal and fatal cardiovascular events [31,36]. This may lie behind the finding that while CVD incidence has been declining in Finland during the past decades [37], the mean BMI has increased significantly [38,39]. MetS irrespective of BMI class may confer increased CVD risk. Even though we do not have the data to study future CVD risk in the present cross-sectional analysis, we have recently shown [40] that the 2009 Harmonization definition of MetS is a significant predictor of future incident CVD and diabetes both in men and in women. In the present study, the Framingham 10-year fatal CVD risk score was significantly lower in individuals without MetS than in those with MetS irrespective of BMI class. As shown by other researchers [8,10,12] and observed in the present study, the metabolically healthy but obese individuals had a slightly less atherogenic lipid profile than normal weight individuals with MetS. However, we did not have data on lipid subclasses or other more detailed biomarkers. No difference was observed in inflammation estimated with the highly sensitive CRP. Longitudinal studies with long enough follow-up periods are needed to reveal the long-term CVD risk related to different obesity phenotypes. However, unlike some earlier data (31, 36), a recent Swedish study with a 30-year follow-up, suggested that increased risk of CVD related to healthy obesity may be detected after only 10 years of follow-up [33].

Different mechanisms behind the different obesity phenotypes include genetic, socioeconomic and behavioral factors, some of which may be modifiable [12,19,20]. In our study, obese individuals without MetS reported similar amount of leisure time physical activity as did the obese individuals with MetS. Contrary to our findings, a US study found leisure time physical activity to be associated with a metabolically healthy obese phenotype [8]. More advanced measures of physical activity may have captured the possible differences in physical activity between the groups in the present study. There were no differences in sleeping patterns between any of the groups. Neither could we detect any differences in consumption of fruit and vegetables. However, more sophisticated methods may be needed to estimate true differences in dietary habits.

To improve comparability of data on healthy obesity, unified criteria for definition of metabolically healthy obesity are urgently needed [30]. These criteria should be suitable for use in population surveys. In the present study we used BMI and the most recent definition of MetS to characterize metabolically healthy obesity. However, BMI does not take into account body composition and amount of body fat. In addition to the need for a definition of healthy obesity, there is a need to develop valid and reliable methods of measuring body composition in population surveys.

The strengths of the present study include a population-based approach and a large and representative sample of middle-aged individuals studied in three districts of Finland. The survey methods have been carefully standardised and comply with international recommendations [22]. However, as previously mentioned, our results are based on cross-sectional data and we cannot determine the future diabetes and CVD risk related to different obesity phenotypes. More sophisticated measures may have captured differences in behavioural factors, but they are labour-intensive to carry out in a population-based survey.

## Conclusions

This cross-sectional population-based study, demonstrated a prevalence of 9-16% of a metabolically healthy phenotype among obese individuals. Metabolic consequences of obesity seem to be more adverse among individuals with MetS. Undetected type 2 diabetes was more prevalent among those with MetS irrespective of BMI class. Increasing BMI had a significantly greater effect on estimates of liver fat and future CVD risk among those with MetS compared with participants without MetS. The healthy obese phenotype was associated with a better overall metabolic profile than that observed in normal weight individuals with MetS.

## List of Abbreviations

(MetS): Metabolic syndrome; (FIN-D2D): the Finnish type 2 diabetes survey; (BMI): body mass index; (HOMA-IR): insulin resistance; (NAFLD): non-alcoholic fatty liver disease; (CVD): cardiovascular disease; (hsCRP): high-sensitivity C-reactive protein.

## Competing interests

The authors declare that they have no competing interests.

## Authors' contributions

EKH, SKK, HO, LN, TS, JTS, JS, MV, MU and MP all had an important role in designing and conducting the FIN-D2D survey. PP wrote the first version of the manuscript. MP participated in the design of the study and performed the statistical analysis. AK, EKH, SKK, HO, LN, TS, JS, MV, MU and MP critically revised the manuscript for important intellectual content. All authors read and approved the final manuscript.

## Pre-publication history

The pre-publication history for this paper can be accessed here:

http://www.biomedcentral.com/1471-2458/11/754/prepub
